# Tritium-Labeled Compounds VII. Isotope Effects in the Oxidation of d-Mannitols-*C*^14^ and d-Mannitols-*t* to d-Fructoses^1^

**DOI:** 10.6028/jres.065A.046

**Published:** 1961-10-01

**Authors:** Lorna T. Sniegoski, Harriet L. Frush, Horace S. Isbell

## Abstract

d-Mannitols, labeled either with carbon-14 at C1, C2, or C3, or with tritium attached to C1, C2, or C3, were prepared. After oxidation by *Acetobacter suboxydans*, the distribution of radioactivity in each of the resulting labeled d-fructoses was determined. Labeled d-mannitol is unique among the hexitols in that it may be oxidized by *A. suboxydans* in either the labeled or the unlabeled part of the molecule. Except in the oxidation of d-mannitol-*2*-*t*, the competing reactions result in the formation of a mixture of d-fructoses, each having radioactivity in one of two different positions. Hence, the isotope effect, k*/k, (where k* and *k* are, respectively, the rate constants for oxidation in the labeled and in the unlabeled part of the labeled d-mannitol molecule) is the ratio of the activities at the two positions of the product, d-fructose.

The following isotope effects were found for the bacterial oxidation of labeled d-mannitols: (1) for d-mannitol-*2*-*C*^14^, k*/k=0.93; (2) for d-mannitol-*2*-*t*, k*/k=0.23; and (3) for d-mannitol-*3*-*t*, k*/k=0.70. For d-mannitols labeled at other positions, no isotope effect was detected, since k*/k was unity. The large isotope-effect for d-mannitol-*2*-*t* is indicative of rupture of the C2–H bond in the rate-determining process. It is suggested that the secondary isotope-effect for tritium at C3 indicates hyperconjugation of the C3 hydrogen atom in the activated enzyme—substrate complex; the lack of such effect for tritium at C1 may be due to unfavorable steric conditions for hyperconjugation of the C1 hydrogen atoms in the complex.

The following substances were prepared and their isotopic distributions determined: d-fructose-*1,6-C*^14^ and d-fructose-*1,6-t* (from 1-labeled d-mannitols); d-fructose-*2,5-C*^14^ and d-fructose-*5-t* (from 2-labeled d-mannitols); and d-fructose-*3,4-C*^14^ and d-fructose-*3,4-t* (from 3-labeled d-mannitols). A procedure, employing d-fructose-*1,6-C*^14^ as an internal standard, was devised for the analysis of d-fructose-*3,4-t.*

## 1. Introduction and Discussion

The burgeoning use of radioisotopes as tracers for studying chemical and biological reactions makes desirable the determination of differences in the behavior of radioactive and nonradioactive atoms. Although the chemical properties of isotopes are essentially the same, certain differences in *rates* of reaction are found, especially when the isotope is directly involved in a rate-determining step [1].^3^ The ratio of the rates of reaction of the labeled and the unlabeled molecules, the isotope effect, is associated with the differences in mass. Carbon-14, the most useful and versatile radioisotope for the study of organic reactions, has a mass 1.167 times that of carbon-12. Isotope effects in the reactions of C^14^-labeled materials are small, and are frequently neglected. However, the radioisotope tritium, having a mass about three times that of hydrogen, produces isotope effects of considerably greater magnitude. Such effects may lead to errors if ignored; nevertheless, they provide a valuable means for studying reaction mechanisms, by indicating whether or not a tritium bond is involved in the rate-determining step of a reaction.

The oxidation of d-mannitol (I) to d-fructose by *Acetobacter suboxydans* is particularly suitable for the study of isotope effects. *A. suboxydans* is highly specific for the oxidation of the penultimate hydroxyl group of any compound having the structure

**Figure:**
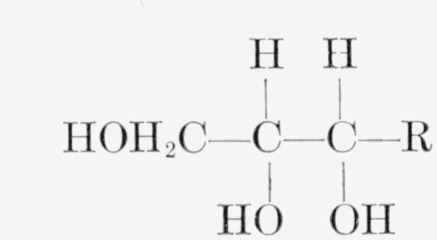

in the d-series [2]. d-Mannitol is unique among the hexitols, because each of the two identical three-carbon portions of the molecule is in the requisite steric arrangement for oxidation by *A. suboxydans.* Thus, in the unlabeled molecule, oxidation can take place equally well at C2 or C5. When one three-carbon portion of the molecule is labeled with either carbon-14 or tritium in any of its three positions, the organism can attack either the labeled or the unlabeled portion. If there is no isotope effect, the product will be an equimolecular mixture of the d-fructoses (II) and (III)^4^, with the radioactivity in the mixture equally divided between two positions. However, if the presence of the radioisotope affects the rate of oxidation, there will be unequal distribution of the radioactivity, a result which can be used for quantitatively evaluating the isotope effect.

**Figure:**
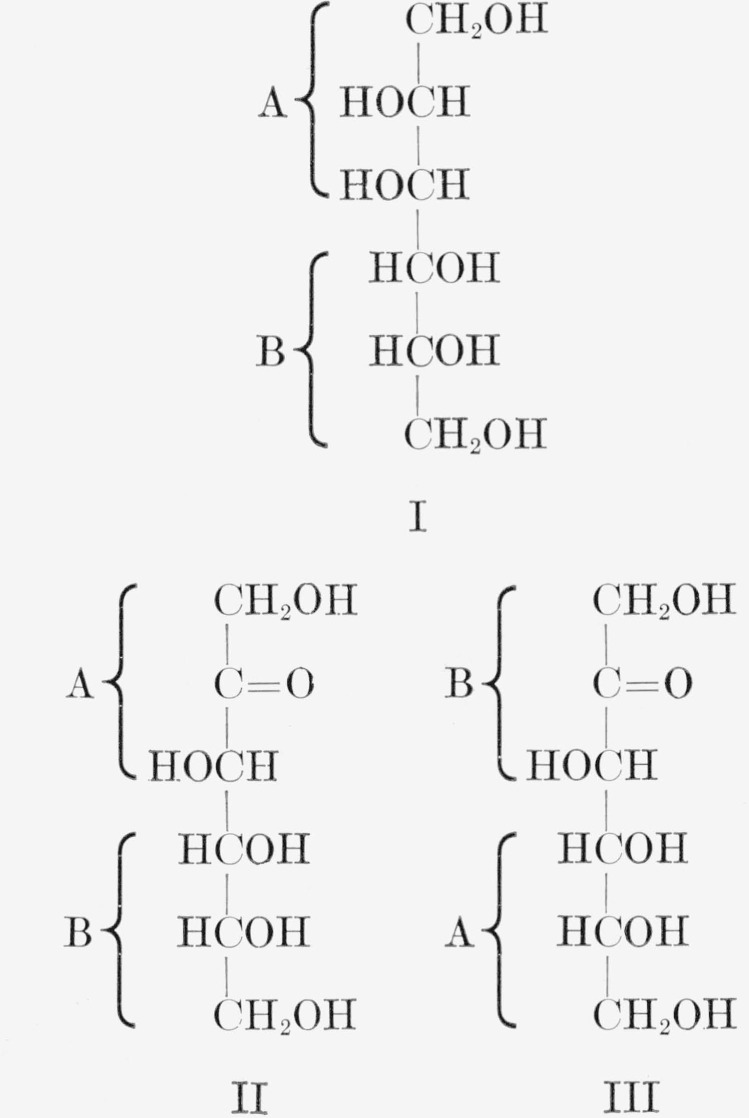


Previously, d-mannitol-*1-C*^14^ had been oxidized by *A. suboxydans* to d-fructose-*1*,*6*-*C*^14^ [3], and the isotopic distribution in the latter compound had been determined [4].^5^ In the present study, d-mannitols labeled either with carbon-14 at C1, C2, or C3, or with tritium attached to C1, C2, or C3, were prepared and were then oxidized by *A. suboxydans.* The resulting labeled d-fructoses were isolated, the isotopic distributions were determined, and the isotope effects were calculated. In the course of the work, methods were developed for determining isotopic distribution; these methods include the use of d-fructose-*1*,*6-C*^14^ as an internal standard in the assay of tritium-labeled d-fructoses.

Tables 1 and 2 summarize the isotope effects determined for the bacterial oxidation of C^14^- and tritium-labeled d-mannitols. It is evident that, in the oxidation of d-mannitols labeled at C2, tritium causes a large isotope effect and carbon-14 a small one. There is also an isotope effect for tritium attached to C3; it is relatively small compared to that at C2, but is larger than the effect of carbon-14 at C2. The oxidation of the remaining three alditols was unaffected by the presence of the isotope. Thus, although *A. suboxydans* has specific steric requirements for the groups attached to a chain of three carbon atoms, an isotope effect is detectable only when carbon-14 is at C2 or when tritium is attached to either C2 or C3.

Although the biological oxidation may be a complicated process, the high yield of a single product suggests an oxidation step that is relatively simple. If the cleavage of the C2—H bond is rate-determining, a large isotope-effect would be expected in the oxidation of d-mannitol-*2*-*t*, and a smaller one in the oxidation of d-mannitol-*2*-*C*^14^. The observed effects are in accordance with this expectation.

The secondary isotope-effect found for tritium at C3 is relatively large, and shows that the C3—H bond is involved, in some manner, in the rate-determining step. The effect may be attributed to hyperconjugation similar to that postulated to explain the effect of *β*-deuterium atoms on the rate of solvolysis of certain deuterated alkyl compounds [5, 6, 7]. Thus, if the C3—H bond of the activated enzyme-substrate complex had partial “no-bond” character, the labeled and unlabeled complexes would differ in the extent of stabilization arising from hyperconjugation. Because a carbon–tritium bond is stronger than a carbon–hydrogen bond, the enzyme-substrate complex labeled with tritium at C3 of the alditol would be less stabilized by hyperconjugation and have greater energy of activation than its unlabeled counterpart. Therefore, in accordance with the experimental results, the rate of reaction would be lower for the labeled than for the unlabeled complex.

Solely on the basis of the above mechanism, there should also be an isotope effect for tritium at C1; this should be less than that at C3, because C1, with tritium attached, bears a hydrogen atom also. The absence of such an effect is surprising and may be indicative of a heretofore unrecognized steric factor in the reaction mechanism. Evidence has been advanced by others that the extent of hyperconjugation depends, to some degree, on the orientation of the C—H bond [7]. Inasmuch as the atoms in the activated enzyme–substrate complex are, presumably, oriented in a definite conformation, it seems possible that steric conditions are favorable for hyperconjugation of the hydrogen atom at C3, but unfavorable at C1.

## 2. Evaluation of the Isotope Effect in the Bacterial Oxidation of Labeled d-Mannitols to Labeled d-Fructoses

The isotope effect causes changes in the isotopic composition of both substrate and product as a reaction proceeds [1, 8]. Evaluation of the effect is complicated by the dependence of the isotopic composition on the extent of the reaction. Labeled molecules of d-mannitol contain *two* possible sites which will compete in the bacterial oxidation; at (or near) one of these sites there is an isotopic atom. Thus, in labeled d-mannitol, the isotope effect is manifested in two different ways, namely, (a) by intermolecular differences in the overall oxidation rates of labeled and unlabeled molecules, and (b) by intramolecular competition for oxidation. The latter isotope effect can be formulated, somewhat arbitrarily, as the ratio k*/k, where k* and *k* are, respectively, the overall rate constants for oxidation of the labeled and unlabeled portions of the labeled molecules of d-mannitol. (See page 47 of ref 1.) This ratio is constant during the reaction and independent of changes in isotopic composition caused by the intermolecular effect.

Because the labeled substrate simultaneously forms two products by competitive reactions, it follows from the law of mass action that

$$P_{A}/P_{B}=k*/k$$ (1)

where *P*_A_ and *P*_B_ are, respectively, the amounts (in millimoles) of the labeled products, d-fructose (II) and d-fructose (III), formed by oxidation at C2 and C5, respectively, of the labeled molecules of d-mannitol. If *n* millimoles of total d-fructose are formed, then (except in the oxidation of d-mannitol-*2-t*)

$${\left[A\right]}_{A}=P_{A}\phi /n$$ (2)

where [*A*]_A_ is the amount of radioactivity of C1, C2, or C3 per millimole of the total product, and *ϕ* is the amount of radioactivity of one milliatom of tritium or carbon-14. Similarly,

$${\left[A\right]}_{B}=P_{B}\phi /n$$ (3)

where [*A*]_B_ is the amount of radioactivity of C4, C5, or C6 per millimole of the total product. It follows from eqs (1), (2), and (3) that

$${\left[A\right]}_{A}/{\left[A\right]}_{B}=P_{A}/P_{B}=k*/k.$$ (4)

In the oxidation of d-mannitols labeled with carbon-14 at C1, C2, or C3, or with tritium attached to C1 or C3, the isotope effect, k*/k, was calculated, by means of eq (4), from values of [*A*]_A_ and [*A*]_B_ determined from the distribution analysis.

In the oxidation of d-mannitol-*2*-*t*, the tritium is removed when oxidation occurs at C2, and the products of the oxidation are nonradio active d-fructose and d-fructose-*5*-*t*. If [*A*]_M_ is the amount of radioactivity per millimole of d-mannitol-*2*-*t*, then the molar activity *lost* at C2 (which is a measure of the oxidation at this point) is [*A*]_M_*—*[*A*]_B_. Hence, from eq (4),

$$\frac{{\left[A\right]}_{M}-{\left[A\right]}_{B}}{{\left[A\right]}_{B}}=P_{A}/P_{B}=k*/k.$$ (5)

The isotope effect in the oxidation of d-mannitol-*2*-*t* was calculated by means of eq(5)^6^ from the molar activities of d-mannitol-*2*-*t* and d-fructose-*5*-*t*.

## 3. Methods for Determining Isotopic Distribution in Labeled d-Fructoses

The procedures devised for the isotopic analysis of the three C^14^-labeled d-fructoses are given in table 1. The general method, common to all three analyses, involves the cleavage of the molecule between C3 and C4. d-Fructose was converted to d-“glucose” phenylosotriazole (IV) which, by oxidation, yielded 4-formyl-2-phenylosotriazole (V), formic acid, and formaldehyde. The molar activity of V is that of the portion of d-fructose including C1, C2, and C3, and the difference between the molar activities of IV and V is the molar activity of the portion including C4, C5, and C6. This method was supplemented by the analysis of C6 of d-fructose-*1,6-C*^14^, and of compounds formed from fragments of d-fructose-*2*,*5-C*^14^.

**Figure:**
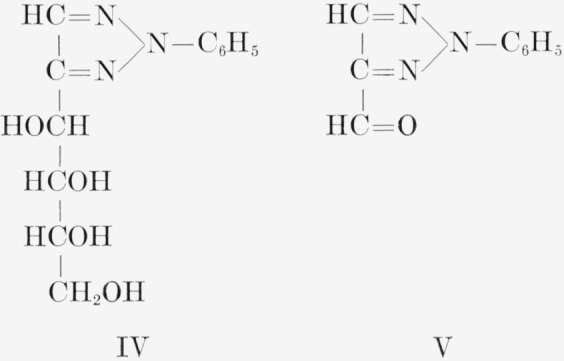


The carbon-14 in d-fructose-*1,6-C*^14^ prepared by the method used here was previously reported by Frush and Isbell [4] to be equally and exclusively distributed between C1 and C6. Their analysis was based on the molar radioactivities of d-fructose- *1,6-C*^14^ and of two oxidation products, namely, potassium d-arabonate-*5-C*^14^ (VI) and formaldehyde- C^14^; the latter was derived from C5 of the potassium d-arabonate-*5*-*C*^14^ and isolated as the dimedon compound (VII). The isotopic distribution of d-fructose-*1,6-C*^14^, determined in the present study by the method given in table 1, is in accord with the previous conclusion.^7^

**Figure:**
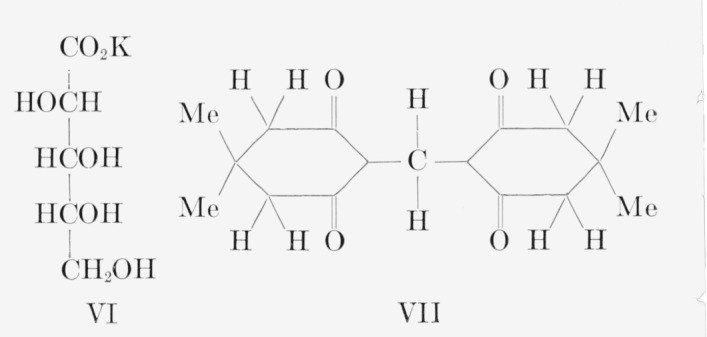


d-Fructose-*2,5-C*^14^ was analyzed by the general method, supplemented by additional procedures given in table 1.^8^ The sugar was degraded to potassium d-arabonate-*1,4-C*^14^, the assay of which was confirmed by that of the corresponding benzimidazole (VIII). Potassium d-arabonate-*1,4-C*^14^ was also degraded to d-erythrose-*3-C*^14^, which was analyzed both as the reduction product, d-erythritol- *3-C*^14^ (IX),^9^ and the corresponding tetrabenzoate.

**Figure:**
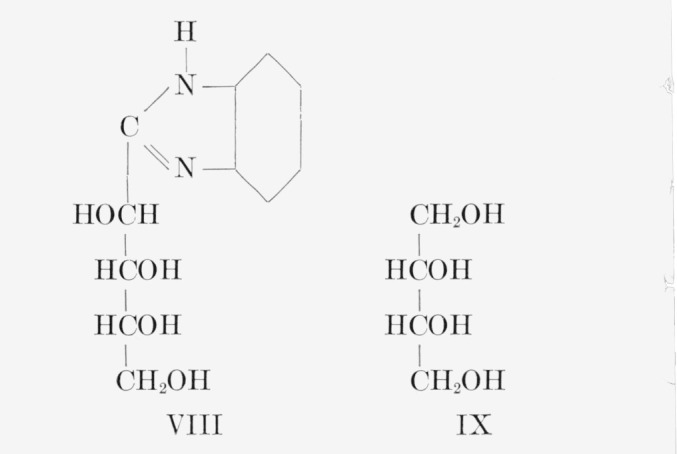


d-Fructose-*3,4-C*^14^ was assayed only by the general method, which determines the radioactivity collectively at the unit of C1, C2, and C3, and at the unit of C4, C5, and C6. This assay was considered adequate, because the prior analysis [4] of d-fructose-*1,6-C*^14^ had shown that there is no redistribution of radioactivity in the molecule by fragmentation and resynthesis.

The procedures used for the isotopic analysis of the three tritium-labeled d-fructoses are outlined in tables 1 and 2. The distribution of tritium in d-fructose-*1,6-t* was determined from the radioassay of this substance and that of the potassium d-arabonate-*5-t* derived from it. The isotope effect in the oxidation of d-mannitol-*2-t* was calculated by means of eq (5) from the relationship between the activity of the d-mannitol-*2-t* and that of the oxidation product, d-fructose-*5-t*.

The isotopic analysis of d-fructose-*3,4*-*t* required the following reactions: oxidation of d-fructose-*3*,*4-t* to potassium d-arabonate-*2,3-t;* degradation of this to d-erythrose-*1,2-t;* and oxidation of d-erythrose- *1,2-t* to d-erythrono-l,4-lactone-*2-t* (X), the product

**Figure:**
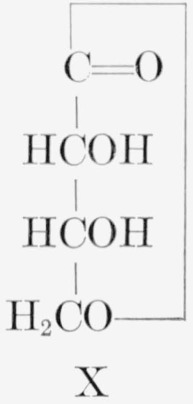

analyzed. Because the yield from this series of reactions was low, a new technique was devised for facilitating the analysis. d-Fructose-*1,6-C*^14^ (which is *equally labeled* at C1 and C6) was added to the d-fructose-*3,4-t*, and this mixture was recrystallized and treated as described above. The isotopic distribution in the d-fructose-*3,4-t* was calculated by the following method.

If the d-fructose-*3,4-t-1,6-C*^14^ contains *P*_A_+*P*_B_ millimoles of tritium-labeled d-fructose, the activity due to tritium is *P*_A_*ϕ*_t_+*P*_B_*ϕ*_t_; where *ϕ*_t_ is the activity of a milliatom of tritium. The activity due to carbon- 14 is 2*P*_F_*ϕ*_c_, where *P*_F_ is the amount (in millimoles) of *either* d-fructose-*1-C*^14^ or d-fructose-*6-C*^14^, and ϕ_c_ is the activity of a milliatom of carbon-14. In the corresponding d-erythrono-1,4-lactone-*2-t-4-C*^14^, the activity due to tritium is *P*_B_*ϕ*_t_, and the activity due to carbon-14 is *P*_F_*ϕ*_C_. Then

$$\rho {}^{\circ}=\frac{P_{A}\phi_{t}+P_{B}\phi_{t}}{2P_{F}\phi_{C}}$$ (6)

and

$$\rho =\frac{P_{B}\phi_{t}}{P_{F}\phi_{C}}$$ (7)

where *ρ*° and *ρ* are, respectively, the tritium-carbon- 14 ratios in the d-fructose and the corresponding d-erythrono-1,4-lactone. It follows from eqs (6) and (7) that

$$P_{A}/P_{B}=(2\rho {}^{\circ}-\rho )/\rho =k*/k.$$ (8)

The isotope effect in the oxidation of d-mannitol-*3-t* was calculated from the analytical data by means of eq (8). This method permits the use of a nonradioactive carrier at any step in the analysis, because the calculation depends only on the *ratios* of tritium to carbon-14.

## 4. Experimental Procedures

### 4.1. Preparation of Labeled d-Mannitols

d-Mannitol-*1-C*^14^ and d-mannitol-*2-C*^14^ were prepared by the sodium borohydride reduction [11] of d-mannono-1,4-lactone-*1-C*^14^ [12] and d-mannono- 1,4-lactone-*2-C*^14^ [13], respectively. d-Mannitol-*1-t* and d-mannitol-*2-t* were prepared by the reduction of d-mannono-1,4-lactone and d-fructose, respectively, with tritiated lithium borohydride [14]. d-Mannitol-*3-C*^14^ was prepared from d-glycerose by three successive cyanohydrin syntheses, in the first of which, sodium cyanide-*C*^14^ was used. The d-erythrono-1,4-lactone-*1-C*^14^ obtained in the first step was converted to d-erythrose-*1-C*^14^ by catalytic reduction of tri-*O*-acetyl- d-erythronyl-*1-C*^14^ chloride [15, 16] and subsequent deacetylation. By methods analogous to those devised for the synthesis of d-mannono-1,4-lactone-*2-C*^14^, d-erythrose-*1-C*^14^ was converted to d-arabinose-*2-C*^14^, and this, in turn, to d-mannono-1,4-lactone-*3-C*^14^. Finally, the lactone was reduced to d-mannitol-*3-C*^14^ [11].

d-Mannitol-*3-t* was prepared by the following series of reactions: d-Arabonic acid $\overset{\text{oxidation}\;[17]}{\underset{\left(V_{2}O_{5}+{\text{NaClO}}_{3}\right)}{\to }}$

$$\begin{array}{l} \underset{\left(2\text{-keto-D-arabonic acid}\right)}{\text{D-}erythro\text{-pentulosonic acid}}\;\overset{\text{reduction}}{\underset{\left({\text{LiBH}}_{4}\text{-}t\right)}{\to }}\\ \{\begin{array}{l} \text{D-arabonic-}2\text{-}t\;\text{acid}\;\to \text{D-arabono-}1,4\text{-lactone-}2\text{-}t\\ \text{D-ribonic-}2\text{-}t\;\text{acid} \end{array}\\ \overset{\text{reduction}}{\underset{\left({\text{NaHg}}_{x}\right)}{\to }}\;\text{D-arabnose-}2\text{-}t\;\overset{\text{eyanohydrin synthesis}}{\underset{\left(\text{NaCN}\right)}{\to }}\\ \text{D-mannono -}1,4\text{-lactone-}3\text{-}t\;\overset{\text{reduction}}{\underset{\left({\text{NaBH}}_{4}\right)}{\to }}\;\text{D-mannitol-}3\text{-}t. \end{array}$$

The experimental details for the preparation of d-mannitol-*3-C*^14^ and d-manmtol-*3-t* will be published in subsequent papers from this laboratory.

### 4.2. Bacterial Oxidation of Labeled d-Mannitols and Preparation of Labeled d-Fructoses

The labeled d-mannitols were oxidized with a culture of *Acetobacter suboxydans*^10^ which was maintained as follows: Agar slants were prepared from 10-ml aliquots of an aqueous solution containing, by weight, 0.5 percent of yeast extract, 0.3 percent of peptone, 1.5 percent of agar, and 5.0 percent of d-mannitol. After being sterilized and cooled, these slants were streaked with *A. suboxydans* and kept at 30 °C for 24 to 48 hr; if not used at once, the organism was stored at 5 to 10 °C and transferred about once a month.

Inoculum for the oxidations was prepared from a broth containing 1 g of yeast extract, 0.6 g of potassium dihydrogen phosphate, and 3.64 g of d-mannitol in 200 ml of aqueous solution. Portions (25 ml) of the broth were measured into 125-ml Erlenmeyer flasks and sterilized. *A. suboxydans* was introduced from a slant, and the flasks were kept at 30 °C. After 48 hr, a satisfactory growth had occurred, and the inoculum was ready for use.

All oxidations of d-mannitol were conducted in 50-ml Erlenmeyer flasks, each containing 1 mmole of d-mannitol, 50 mg of yeast extract, 30 mg of potassium dihydrogen phosphate, and 10 ml of water. The solutions were sterilized and cooled. Five drops of inoculum were added to each solution with a sterile pipet, and the cultures, after gentle mixing, were kept at 30 °C in an incubator.

Oxidations were performed first with unlabeled d-mannitol under the conditions just described, in order to determine the time required for optimal yields of d-fructose. At suitable time-intervals, the preparations were deproteinized [18] by the addition of 1 ml of a 0.7-*M* aqueous solution of zinc sulfate, and neutralization (to the phenolphthalein end-point) with a saturated aqueous solution of barium hydroxide. The precipitate was removed by filtration through a layer of paper pulp and purified diatomaceous earth, the filtrate was diluted to 100 ml in a volumetric flask, and 1-ml aliquots were analyzed for total reducing-substance^11^ by the Somogyi method [19].

Oxidations of the *labeled* d-mannitols were then conducted by the above procedure. Simultaneously with a labeled material, nonradioactive controls were oxidized, and the progress of the oxidation was followed by analysis of the controls. When the total reducing-substance in the controls was approximately the same as the maximum found previously, the product from the labeled d-mannitol was deproteinized, filtered, de-ionized by passage through mixed cation- and anion-exchange resins,^12^ and finally freeze-dried. The residue was dissolved in 2 ml of anhydrous methanol containing a drop of acetic acid, and the solution was filtered through decolorizing carbon into a standard-tapered test tube and concentrated at 30 °C under diminished pressure.^13^ Addition of methanol and evaporation were repeated several times; finally, the sirup was dissolved in methanol, 2-propanol was added to incipient turbidity, and the solution was nucleated with crystalline d-fructose. Additional 2-propanol was added from time to time, as the crystallization proceeded. The mother liquor was removed from the crystals with a capillary pipet, and the crystals were washed with a mixture of methanol and 2-propanol and dried in a vacuum desiccator. The labeled d-fructose was recrystallized from methanol by the addition of 2-propanol. Additional crops were obtained from the mother liquor by the use of nonradioactive d-fructose as a carrier. The total radiochemical yield was 70 to 75 percent.

In the oxidation of d-mannitol-*2-t*, it was necessary to avoid the introduction of unlabeled d-mannitol with the inoculum, and also to isolate and purify the d-fructose-*5-t* without carrier, because the isotopic distribution was determined from the relative molar activities of these two labeled compounds. For this oxidation, the inoculum was treated as follows: A 25-ml portion was centrifuged, and the supernatant liquor was decanted from the precipitated bacteria. The precipitate was mixed with 10 ml of a sterile, 0.15-*M* aqueous solution of potassium chloride, and the suspension was centrifuged. The liquid was again decanted, and the rinsing process was repeated. Finally, the bacteria were suspended in 20 ml of the potassium chloride solution, and each of the solutions containing d-mannitol-*2-t* was inoculated with five drops of this suspension. The remainder of the procedure was the same as that used with the other labeled d-mannitols.

Before analysis, the d-fructoses were chromatographically pure, as indicated by radioautographs or scans of chromatograms developed in 1-butanol–acetic acid–water (4:1:5 v/v, upper phase) and in 2-butanone–acetic acid–water saturated with boric acid (9:1:1) [20]. Each labeled d-fructose except d-fructose-*5-t* was diluted with nonradioactive d-fructose to a specific activity convenient for the analysis, and the mixture was recrystallized. The C^14^-labeled d-fructoses used for analysis had activities of about 0.01 *μ*c/mg, and the tritium-labeled d-fructoses, 0.2 to 0.3 *μ*c/mg. For the isotopic analysis of d-fructose-*3,4-t*, a mixture of this compound and d-fructose-*1,6-C*^14^ was prepared, recrystallized, and analyzed for both tritium and carbon-14 (see table 2 and section 3).

### 4.3. Preparation of Compounds Used in the Isotopic Analysis of Labeled d-Fructoses

#### 4.3.1. “d-Glucose” Phenylosotriazole (IV) [21, 22]

A solution of the labeled d-fructose (250 mg, 1.39 mmole) in 10 ml of water, contained in a 50-ml, round-bottomed flask, was treated with one drop of acetic acid and 0.45 ml (4.56 mmole) of phenylhydrazine, and allowed to stand at room temperature for 20 min. Sodium acetate trihydrate (250 mg) and acetic acid (0.5 ml) were then added, and the solution was heated in a boiling-water bath for 30 min, treated with 5 ml of water, and kept at about 5 to 10 °C for 2 hr. Crystalline d-glucose phenylosazone was remove by filtration and washed with a little water. The osazone, transferred to a 200-ml round-bottomed flask, was refluxed for 1 hr with 30 ml of 2-propanol and a solution of 1 ml of 6-*N* sulfuric acid and 1.5 g of copper sulfate pentahydrate in 45 ml of water. The hot mixture was filtered through a layer of purified diatomaceous earth and decolorizing carbon, and the filtrate was concentrated under diminished pressure to about 15 ml. The triazole, which crystallized readily, was separated by filtration, washed with water, and dried in a vacuum desiccator; the yield was about 75 mg (20%). After three or four recrystallizations from ethanol,^14^ the radioactivity was constant; the molecular radioactivity serves as a check on that of the original labeled d-fructose.

#### 4.3.2. 4-Formyl-2-phenylosotriazole (V) [21]

Labeled d-glucose phenylosotriazole (80 mg, 0.30 mmole) and sodium metaperiodate (277 mg, 1.3 mmole) in 12 ml of water, in a glass-stoppered testtube, were mechanically shaken for 24 hr, during which time the appearance of the crystals changed. The crystalline product, 4-formyl-2-phenylosotriazole, was separated and dissolved in the minimal amount of warm ethanol; after filtration through decolorizing carbon, the solution was concentrated to a small volume under a stream of nitrogen, and treated with water to incipient turbidity. When crystallization was complete, the compound was separated, washed with ice water, and dried in a desiccator. The yield, after two recrystallizations from warm ethanol by the addition of water, was 65 to 70 percent, and the specific radioactivity was constant.

#### 4.3.3. Potassium d-Arabonate (VI) [4]

A solution of labeled d-fructose (360 mg, 2 mmoles) in 10 ml of water was frozen on one part of the wall of a heavy-walled flask, and 10 ml of 2-*M*, aqueous potassium hydroxide was frozen on another part. The flask was immediately attached to a Parr apparatus, evacuated, filled with oxygen at a pressure of 10 psi, and shaken for 24 hr at room temperature. The solution was then diluted with 300 ml of methanol, nucleated with a minute quantity of crystalline potassium d-arabonate, and stored in the refrigerator for 24 hr. The mother liquor was decanted from the crystals, which adhered to the wall of the flask, and the crystals were washed with methanol and dissolved in a few milliliters of water. The solution was filtered into a standard-tapered test-tube, concentrated in a stream of nitrogen to about 3 ml, and diluted with methanol to incipient turbidity. After crystallization was complete, about 260 mg, or 64 percent, of potassium d-arabonate had separated. This was slowly recrystallized several times by storing an aqueous solution of the material in a desiccator containing anhydrous calcium sulfate and a beaker of methanol. Large, pure crystals separated in the course of 1 or 2 days. The activity was constant after three recrystallizations. The molar activity was checked by the preparation and assay of the derived benzimidazole.

#### 4.3.4. Benzimidazole From d-Arabonic Acid (VIII) [23]

Potassium d-arabonate (90 mg, 0.44 mmole) was combined in a test-tube with *o*-phenylenediamine hydrochloride (80 mg, 0.44 mmole), and 0.3 ml of a solution prepared from 4 ml of water, 1 ml of ethanol, and 0.85 ml of concentrated hydrochloric acid. The mixture was heated in an oil bath at 135 °C for 2 hr; during the first hour, 50-percent aqueous ethanol was added, at intervals, in order to prevent evaporation to dryness. Finally, the residue was dissolved in 0.5 ml of water, and the solution was filtered through decolorizing carbon. When the filtrate and washings were diluted with water to about 1.5 ml and made just alkaline with dilute ammonium hydroxide, crystallization of the derivative occurred. The crystals were separated and washed, successively, with ethanol, acetone, and ether. The benzimidazole derivative (about 92 mg or 87%) was recrystallized once from hot methyl Cellosolve (2-methoxyethanol) and once from 50-percent, aqueous ethanol.

#### 4.3.5. d-Erythritol-*3-C*^14^ (IX)

Potassium d-arabonate-*1,4-C*^14^ (300 mg, 1.47 mmoles, prepared from d-fructose-*2,5-C*^14^) was dissolved in 7.5 ml of water, and 0.3 ml of an aqueous solution of barium acetate (9 g/100 ml), 0.3 ml of an aqueous solution of ferrous sulfate (9.2 g of heptahydrate/100 ml), and 0.15 ml of 30-percent hydrogen peroxide were added [24]. The mixture was kept at 45 °C for 90 min, again treated with 0.15 ml of hydrogen peroxide, and allowed to stand at 45 °C for another hour. The mixture was then filtered through a small amount of decolorizing carbon, and the filtrate was passed through 15 ml of mixed cation- and anion-exchange resins. The effluent, containing d-erythrose-*3-C*^14^, was concentrated under diminished pressure to a sirup.

A solution of the sirup in 15 ml of water was cooled in an ice bath and stirred with a magnetic stirrer. Freshly prepared, 0.3-*M*, aqueous sodium borohydride (30 ml) was added dropwise from a buret, and stirring of the ice-cold solution was continued for 90 min. A small amount of cation-exchange resin was then added, and the solution was passed through a column containing 15 ml of the resin; the effluent was concentrated under diminished pressure to a thin sirup. In order to remove boric acid as methyl borate, the sirup was dissolved in methanol, and the solvent was distilled under diminished pressure; after this process had been repeated several times, the sirup crystallized. The yield of crude d-erythritol-*3-C*^14^ was 124 mg (69%).

A solution of the crude material in ethanol and water was filtered through decolorizing carbon and concentrated under diminished pressure to a sirup. The d-erythritol-*3-C*^14^ was then recrystallized to constant activity from the minimal amount of hot ethanol by the addition of 2-propanol to incipient turbidity. The molar radioactivity was checked by the preparation and radioassay of d-erythritol-*3-C*^14^ tetrabenzoate.

#### 4.3.6. D-Erythritol-*3-C*^14^ Tetrabenzoate

A solution of d-erythritol-*3-C*^14^ (80 mg, 0.66 mmole) in 2 ml of pyridine, contained in a 50-ml flask having a magnetic stirrer, was cooled in an ice bath. Benzoyl chloride (1 ml, 8.6 mmoles) was added, and the mixture was stirred in the ice bath for several hours, allowed to stand overnight at room temperature, and treated with ice water which precipitated a sirup; this was successively washed with water, 5-percent aqueous sodium carbonate, and water. Upon addition of ethanol, the simp crystallized; the crystals were separated and recrystallized to constant radioactivity from methyl Cellosolve.

#### 4.3.7. Formaldehyde–Dimedon Compound (VII) [4]

A mother liquor, that remained after a preparation of 4-formyl-2-phenylosotriazole from d-fructose-*1,6-C*^14^ by the method of section 4.3.2, theoretically contained about 0.5 mmole of C^14^-labeled formaldehyde in 20 ml of water. An aqueous solution of sodium bisulfite was added dropwise to this solution, in an amount just sufficient to remove the iodine that first appeared. (As the end-point was approached, a dilute solution of the bisulfite was used.) The mixture was then neutralized (to the end-point of methyl orange) by the dropwise addition of a solution of sodium bicarbonate. To the reaction mixture was added 5 ml of an aqueous solution containing 182 mg of sodium bicarbonate and 182 mg (1.30 mmole) of dimedon (5,5-dimethyl-1, 3-cyclohexanedione). The formaldehyde–dimedon compound crystallized readily; it was separated, washed with water, and recrystallized by dissolving it in the minimal amount of hot methyl Cellosolve, filtering the solution through decolorizing carbon, and adding an equal volume of water. Recrystallization was repeated until the specific radioactivity was constant.

#### 4.3.8. d-Erythrono-1,4-Lactone-*2-t-4-C*^14^ (X)

d-Fructose-*3,4*-t-*1,6-C*^14^ (181 mg, 1.0 mmole having a tritium/carbon-14 ratio of 7.46) was degraded by the procedure described in section 4.3.3 to yield 144 mg (70.2%) of potassium d-arabonate-*2,3-t-5-C*^14^. A 133-mg sample of this compound was degraded to d-erythrose-*1,2-t-4-C*^14^ by the procedure of section 4.3.5. The sirupy d-erythrose (39 mg, 0.33 mmole) so obtained was dissolved in 1.5 ml of water, and the solution was cooled in an ice bath. Barium benzoate dihydrate (215 mg, 0.52 mmole) and bromine (0.020 ml, 0.39 mmole) were added, and the mixture was shaken until all of the bromine had dissolved; it was then allowed to stand overnight at room temperature [25]. Excess bromine was removed by the addition of a small amount of decolorizing carbon, and the mixture was filtered. To the filtrate was added 122 mg (0.30 mmole) of nonradioactive barium d-erythronate. An aqueous solution of silver sulfate (156 mg, 0.5 mmole) was added, and the precipitate of silver bromide and barium sulfate was removed by filtration. The solution was then passed through a column of cation-exchange resin (10 ml), the effluent extracted with chloroform, and the aqueous solution concentrated under reduced pressure to a sirup. Absolute ethanol was added, and the solution was again concentrated; this process was repeated several times. Finally, the sirup was moistened with absolute ethanol, nucleated with d-erythrono-1,4-lactone, and stored in a desiccator for several days, during which time, crystallization of d-erythrono-1,4-lactone-*2-t-4-C*^14^ occurred. The compound (74 mg) was recrystallized from absolute ethanol.

In order to obtain d-fructose-*3,4-t-1,6-C*^14^ at a level of activity suitable for radioassay, a portion of the d-fructose-*3,4*-*t-1,6-C*^14^ used in the oxidative degradation was diluted with the unlabeled compound and recrystallized. Both the d-fructose-*3,4-t-1,6-C*^14^ and the d-erythrono-1,4-lactone-*2-t-4-C*^14^ were analyzed by the method described in section 4.4, and, from the tritium/carbon-14 ratios (*ρ*° and *ρ*, respectively), k*/k was calculated by eq (8). The results are given in table 2.

### 4.4 Radioassay of Carbon-14 and Tritium

All measurements of radioactivity were made with a windowless, gas-flow, proportional counter by methods described in prior publications from this laboratory. The same procedures, equipment, and reagents were used in the determination of the molar activities of any group of compounds which were to be compared.

Samples containing carbon-14 (only) were dissolved in formamide, *N,N*-dimethylformamide, or ethylene glycol, and counted from a layer of solution that was “infinitely thick” to the radiation [26]; in each individual assay, the sample was counted to at least 10,000 counts. The activities of the C^14^- labeled compounds were calculated from the relationship:

A=amk (9)

where *A* is the activity, in microcuries, of the sample counted; *a*, the observed counts per second (cps) corrected for background; *m*, the combined weight, in grams, of the solute and solvent; and *k*, an empirically determined constant, which, with the equipment used, was 2.39×10^−3^ *μ*c cps^−1^ g^−1^. The calculation is illustrated by the following: A 9.625- mg sample of a compound having a molecular weight of 265.3 was dissolved in 1 ml (1.133 g) of formamide; the solution gave 31.48 cps. The activity in the sample was 31.48×1.143×2.39×10^−3^, or 0.0860 *μ*c, corresponding to (0.0860/9.625)×265.3 or 2.37 *μ*c/mmole.

Samples containing tritium (only) were assayed in the proportional counter in films that were infinitely thick to the radiation [27]. In each assay, five films, from aliquots of the same solution, were prepared on planchets; each film was counted to at least 10,000 counts, and the average value was used. Activities of tritium-labeled compounds were also calculated by means of eq (9). For these compounds, *A* is the activity, in microcuries, of the film; *a* is the observed cps; *m* is the weight, in milligrams, of the film; and *k*, with the equipment used, is 4.45×10^−5^ *μ*c cps^−1^ mg^−1^. Thus, a 0.5060- mg sample of a compound with a molecular weight of 182.2, in a film weighing 20.22 mg, gave 123.27 cps. The activity in the sample was 123.27×20.22 ×4.45×10^−5^, or 0.1109 *μ*c; this corresponds to (0.1109/0.5060) × 182.2, or 39.9 *μ*c/mmole.

Samples containing *both* tritium and carbon-14 were also assayed in films [28]. The films were counted both with and without a screen of 1/4-mil, double-aluminized Mylar,^15^ which completely stops the radiation from tritium, but admits a portion of that from carbon-14 to the sensitive area of the counting chamber. By means of empirically determined counting-efficiencies, the amount of carbon-14 was calculated from the difference between the counts without and with the screen.

The activity of carbon-14 in the sample is given by the relationship:

$$A_{C}=\frac{a^{\prime }}{3.7\times 10^{4}{E^{\prime }}_{m}}$$ (10)

where *A_C_* is the radioactivity, in microcuries, of the carbon-14 in a film of weight *m*, and *E^′^_m_* and *a′*, respectively, are the counting efficiency of carbon-14 and the cps observed, both with the screen in place; 3.7×10^4^ is the disintegration rate (in. dps) per microcurie. The activity of tritium in the sample is calculated from the following relationship, in which allowance is made for the radiation of carbon-14:

$$A_{t}=mk[a-(a^{\prime }E_{m}/{E^{\prime }}_{m}].$$ (11)

*A_t_* is the radioactivity, in microcuries, of the tritium in the film; *m* is the weight, in milligrams, of the film; *k* is an empirically determined constant (1.90×10^−4^ *μ*c cps^−1^ mg^−1^); and *E_m_* and *a*, respectively, are the counting efficiency of carbon-14 and the cps observed, both without the screen in place.

The calculation of the two activities in the mixture is illustrated by the use of typical data on the assay of d-fructose-*3,4-t-1,6-C*^14^. A 1.985-mg sample in a film-forming solution gave a film weighing 4.93 mg, for which *a*′ was 37.59 cps, *a* was 124.51 cps, *E^′^_m_* was 0.184, and *E_m_* was 0.395. *A_c_*= 37.59/(3.7×10^4^×0.184)=0.00552 *μ*c. The specific activity with respect to carbon-14 is 0.00552/1.985 = 0.00278 *μ*c/mg. *A_t_*=4.93×1.90×10^−4^[124.51−37.59 (0.395/0.184)1 = 0.0409 *μ*c. The specific activity with respect to tritium is 0.0409/1.985 = 0.0206 *μ*c/mg.

## Figures and Tables

**Table 1:** Isotope effect in the bacterial oxidation of five position-labeled d-mannitols; analysis of isotopic distribution in the resulting d-fructoses

	Carbon atoms of original d-fructose^a^	Average fraction of the activity of original d-fructose, %	Isotope effect, k^*^/k, in d-mannitol oxidation

d-Mannitol-*1*–*C*^14^			

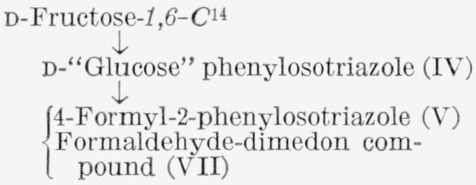	1*, 2, 3, 4, 5, 6	}	
	1, 2, 3, 4, 5, 6*		
	1*, 2, 3, 4, 5, 6	} 100.0	
	1, 2, 3, 4, 5, 6*		
	1*, 2, 3	49.8	} 0.99
	6*	50.1	

d-Mannitol-*2*-*C*^14^			

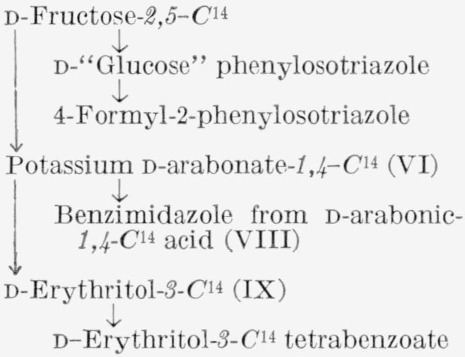	1, 2*, 3, 4, 5, 6	$\begin{array}{r} \}\\ \} \end{array}\}\;100.0$	
	1, 2, 3, 4, 5*, 6		
	1, 2*, 3, 4, 5, 6		
	1, 2, 3, 4, 5*, 6		
	1, 2*, 3	48.4	} 0.93
	2*, 3, 4, 5, 6	$\begin{array}{r} \}\\ \} \end{array}\}\;\left(100.0\right)$	
	2, 3, 4, 5*, 6		
	2*, 3, 4, 5, 6		
	2, 3, 4, 5*, 6		
	3, 4, 5*,6	} 52.1	
	3, 4, 5*, 6		

d-Mannitol-*3*-*C*^14^			

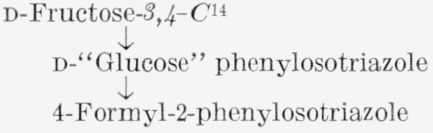	1, 2, 3*, 4, 5, 6	$\begin{array}{r} \}\\ \} \end{array}\}\;100.0$	} 0.99
	1, 2, 3, 4*, 5, 6		
	1, 2, 3*, 4, 5, 6		
	1, 2, 3, 4*, 5, 6		
	1, 2, 3*	49.7	

d-Mannitol-*1*-*t*			

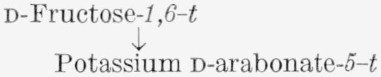	1*, 2, 3, 4, 5, 6	} 100.0	} 1.02
	1, 2, 3, 4, 5, 6*		
	2, 3, 4, 5, 6*	49.5	

d-Mannitol-*2*-*t*			

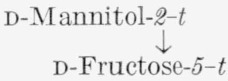	1,2*,3, 4, 5, 6	} 100.0	} 0.23
	1, 2, 3, 4, 5*, 6		
	1, 2, 3, 4, 5*, 6	81.6	

a Position of radioactive atom indicated by*.

**Table 2:** Isotope effect in the bacterial oxidation of d-mannitol-3–*t*; use of the “double-label” technique in the analysis of isotopic distribution in d-fructose-3,4–*t*

Compound analyzed	Radioactivity after successive recrystallizations		Ratio *t*/*C*^14^	Isotope effect, k*/*k*, in d-mannitol oxidation^a^
	*t*	*C*^14^		
				
	*μc/mg*	*μc/mg*		
d-Fructose-*3,4*-*t*-*1,6*-*C*^14^	0.0210	0.00278		
	0.0207	0.00281		
Avg	0.0209	0.00280	7.46	} 0.70
d-Erythrono-1,4-lactone-*2-t-4*-*C*^14^ (X).	0.0253	0.00296		
	0.0258	0.00288		
Avg	0.0256	0.00292	8.76	

a k*/*k* = (2*ρ*°–*ρ*)/*ρ*.

## Footnotes

3 Figures in brackets indicate the literature references at the end of this paper.

4 Unit A in formulas II and III is derived from the same part of the d-mannitol molecule; the same is true of unit B.

5 In previous papers from this laboratory, the alternative designation of the position of the label in a labeled d-mannitol was given in parentheses, e.g., d-mannitol-*1*(*6*)-*C*^14^. In this and subsequent papers in the series, the parenthetical number will be omitted. The oxidation product of d-mannitol-*1*-*C*^14^ contains two singly-labeled species, namely, d-fructose-*1*-*C*^14^ and d-fructose- *6-C*^14^, and is designated d-fructose-*1,6*-*C*^14^ (see formulas I, II, and III).

6 The isotope effect in the oxidation of five of the six labeled d-mannitols used in this study can be determined from the ratio of the activity at two positions in the total product. However, during the oxidation of d-mannitol-*2*-*t*, the intermolecular isotope effect causes alteration in the activity of the d-fructose-*5*-*t* because of the more rapid oxidation of the unlabeled d-mannitol. Therefore, eq (5) applies only at the completion of the reaction, at which time the intermolecular isotope-effect disappears.

7 Brice and Perlin [9], also, have studied the distribution of carbon-14 in d-fructose-*1*,*6-C*^14^ and have found “no significant contamination of the inactive carbon atoms with C^14.”^

8 A brief summary of the analysis of d-fructose-*1*,*6-C*^14^ and d-fructose-*2,5*-*C*^14^ is given in ref 10.

9 This erythritol is asymmetric because of the isotope at C3.

10 Obtained from Dr. N. K. Richtmyer, National Institutes of Health, Bethesda, Md.

11 In their preparation of d-fructose-*1,6-C*^14^, Isbell and Karabinos [3] determined both the total reducing-substance and the d-fructose (the latter by polarization at two temperatures). They showed that the maxima for the two occur at approximately the same time. In the present study, oxidations were stopped when the total reducing-substance was at a maximum (80 percent). 'The time required for the oxidation was usually about 45 hr.

12 Amberlite IR 120-H, Rohm and Haas Co., Philadelphia, Pa., and Duolite A4, Chemical Process Co., Redwood City, Calif. The effluent was tested for ionic impurities by means of a conductivity meter (supplied by Barnstead Still and Sterilizer Co., Boston, Mass).

13 Evaporations in test tubes are conveniently carried out in a Rotary Evapomix, a product of Buchler Instruments, New York, N.Y.

14 The semimicro recrystallizations carried out in this study were conducted in small test-tubes. In some cases, the mother liquor was removed, and the crystals were washed, in place, by means of a capillary pipet.

15 Supplied by Hastings and Co., Inc., Philadelphia, Pa.
